# Integration of Transcriptome and MicroRNA Profile Analysis of iMSCs Defines Their Rejuvenated State and Conveys Them into a Novel Resource for Cell Therapy in Osteoarthritis

**DOI:** 10.3390/cells12131756

**Published:** 2023-06-30

**Authors:** Vasileios Konteles, Ioanna Papathanasiou, Maria Tzetis, Evgenios Goussetis, Varvara Trachana, Evanthia Mourmoura, Charalampos Balis, Konstantinos Malizos, Aspasia Tsezou

**Affiliations:** 1 Laboratory of Cytogenetics and Molecular Genetics, Faculty of Medicine, University of Thessaly, 41110 Larissa, Greece; kontelesb@gmail.com (V.K.); iopapat@uth.gr (I.P.);; 2 Department of Biology, Faculty of Medicine, University of Thessaly, 41110 Larissa, Greece; vtrachana@uth.gr; 3 Department of Medical Genetics, Medical School, National and Kapodistrian University of Athens, 11527 Athens, Greece; mtzetis@med.uoa.gr; 4 Stem Cell Transplant Unit, Aghia Sophia Children’s Hospital, 11527 Athens, Greece; evgoussetis@gmail.com; 5 Department of Orthopaedics, Faculty of Medicine, University of Thessaly, 41110 Larissa, Greece

**Keywords:** iMSCs, miR-arrays, RNA-seq, osteoarthritis, cell therapy

## Abstract

Although MSCs grant pronounced potential for cell therapies, several factors, such as their heterogeneity restrict their use. To overcome these limitations, iMSCs (MSCs derived from induced pluripotent stem cells (iPSCs) have attracted attention. Here, we analyzed the transcriptome of MSCs, iPSCs and iMSCs derived from healthy individuals and osteoarthritis (OA) patients and explored miRNA-mRNA interactions during these transitions. We performed RNA-seq and gene expression comparisons and Protein-Protein-Interaction analysis followed by GO enrichment and KEGG pathway analyses. MicroRNAs’ (miRNA) expression profile using miRarrays and differentially expressed miRNA’s impact on regulating iMSCs gene expression was also explored. Our analyses revealed that iMSCs derivation from iPSCs favors the expression of genes conferring high proliferation, differentiation, and migration properties, all of which contribute to a rejuvenated state of iMSCs compared to primary MSCs. Additionally, our exploration of the involvement of miRNAs in this rejuvenated iMSCs transcriptome concluded in twenty-six miRNAs that, as our analysis showed, are implicated in pluripotency. Notably, the identified here interactions between hsa-let7b/i, hsa-miR-221/222-3p, hsa-miR-302c, hsa-miR-181a, hsa-miR-331 with target genes *HMGA2*, *IGF2BP3*, *STARD4*, and *APOL6* could prove to be the necessary tools that will convey iMSCs into the ideal mean for cell therapy in osteoarthritis.

## 1. Introduction

Osteoarthritis (OA) remains nowadays the most common age-related degenerative joint disease and a major cause of chronic pain and disability. Chondrocyte apoptosis, inflammation, senescence, and extracellular matrix degradation lead to a cascade of catabolic events resulting in the degradation of all joint tissues and, eventually, joint failure [1,2]. Several stem-cell-based strategies have been employed to identify a suitable cell population to regenerate the damaged OA articular cartilage. However, until today there is no effective clinical application [3,4].

Mesenchymal stromal cells (MSCs) are multipotent cells residing in various tissues, including the bone marrow, infrapatellar fat pad, adipose tissue, umbilical cord Wharton’s jelly etc., capable of differentiating into different cell populations of mesodermal origins, such as osteoblasts, adipocytes, and chondrocytes. Due to their high proliferative and self-regenerative ability and low immunogenicity, bone marrow-derived mesenchymal stromal cells (BM-MSCs) have been used in multiple clinical trials in OA [5]. Recent evidence indicates that the cartilage microenvironment influences MSCs’ basic properties, including differentiation, proliferation, and paracrine signalling [6]. Even more, MSC populations are highly heterogeneous, depending on the donor, the tissue of origin and the culture conditions, limiting their large-scale production in genetically stable and reproducible cell lines [7,8].

Ιnduced pluripotent stem cells (iPSCs) have attracted ample attention and have been proposed as a promising cell population for cartilage regeneration in OA since their epigenetic and transcriptional memory has been reported to be erased due to their production process [9,10]. However, further studies suggested that iPSCs retain epigenetic memory of their cellular origin due to residual DNA methylation and histone modification patterns at lineage-specific genes [11,12]. In addition, it was very recently reported that iPSCs from OA patients retained changes in epigenetic factors associated with the OA phenotype of chondrocytes, as well as their transcriptional signature associated with a healthy or osteoarthritic (OA) phenotype depending on the origin of donor cells, posing thus limitations in their regenerative capacity of damaged OA cartilage [13]. iPSCs have been recently used as a cell source for creating functional MSCs (iMSCs), displaying phenotypic similarities with primary MSCs [6,14]. iMSCs are a highly homogeneous cell population exhibiting an MSC-like morphology, expressing key MSC surface markers and mesenchymal genes, while pluripotency-associated genes were strongly down-regulated [14,15,16,17,18]. It has been reported that during reprogramming, cells undergo rejuvenation with augmented cellular vitality demonstrated by enhanced survival, proliferation, and differentiation potential [14,15]. However, studies investigating the chondrogenic potential of iMSCs produced contradictory results. Gusso et al. (2013) revealed that iMSCs have limited culture heterogeneity compared to the iPSCs population. In addition, in micromass culture, they can differentiate into articular chondrocytes characterized by increased *COL2A1* and decreased *COL1A1* expressions and lack of hypertrophic markers [19].

In contrast, Sfougiataki et al. (2020) reported that the differentiation of iMSCs toward chondrocyte micromasses led to an incomplete chondrogenesis process [20]. Moreover, it was reported that iMSCs have less adipogenic and chondrogenic differentiation capacity but are markedly efficient in osteogenesis [16,21,22]. Also, recent studies showed that iMSCs, despite their MSC-like features, are not identical to primary MSCs [23,24]. Furthermore, evidence suggests that iMSCs transcriptome closely resembles that of MSCs, but there are marked differences in their gene expression profile, demonstrating that they represent a distinct cell population [21,22,23,24]. More specifically, Spitzhorn et al. showed that iMSCs, regardless of donor age and cell source, acquired a rejuvenation gene signature that resembles iPSCs but not primary MSCs [24]. Recently, Lee et al. reported distinct characteristics of iMSCs associated with common changes in transcriptomic and proteomic profiles between iMSCs and primary MSCs.

It is known that gene expression and, subsequently, the transcriptome profile of cells are regulated by a complex system that includes transcriptional and epigenetic factors [25]. MicroRNAs (miRNAs), a group of small non-coding RNAs, regulate gene expression post-transcriptionally through mRNA degradation or translation inhibition [26,27]. miRNAs are considered key regulators of self-renewal ability, pluripotency maintenance, proliferation, and differentiation potential of stem cells through directly targeting pluripotency and transcriptional factors implicated in the above processes [28,29]. Moreover, miRNAs have emerged as significant modulators in cell reprogramming, including iPSC generation [29,30,31]. Direct transfection of specific miRNA clusters was sufficient to convert differentiated cells to a pluripotent state, producing thus miRNA-induced iPSCs with high reprogramming efficiency and low tumorigenicity [29]. However, it remains unclear whether the miRNA profile alters during cell reprogramming, which could be associated with transcriptome changes observed in the transition from MSCs-to iPSCs-to iMSCs. The current study aimed to analyze BM-MSCs, iPSCs and iMSCs transcriptome defining iMSCs properties and investigate specific miRNA-mRNA interactions during these cellular transitions.

## 2. Materials and Methods

### 2.1. Bone Marrow Samples

Bone marrow samples were obtained from two (2) young, healthy individuals and five (5) patients with osteoarthritis (OA). Healthy individuals (one female, and one male, ages six and 13 years) were cell transplant donors at Bone Marrow Transportation Unit, Aghia Sophia Children’s Hospital). Bone marrow aspirate was obtained from the medial segment of the iliac crest. The study protocol was approved by the ethical committee of Aghia Sophia Children’s Hospital, Athens, Greece. In osteoarthritis (OA) patients (three females and two males, ages 69, 71, 76, 69 and 79 years), bone marrow aspirate was obtained from the proximal metaphysis of the femur after femoral neck osteotomy before performing hip replacement surgery at the Orthopaedics Department of the University Hospital of Larissa. Radiographs were obtained before surgery and were graded using the Kellgren-Lawrence system. All OA patients had Kellgren-Lawrence grade ≥ 3. The assessment of the radiographs by two independent expert observers was blinded. The study protocol conformed to the ethical guidelines of the 1975 Declaration of Helsinki as reflected in a priori approval by the local ethical committee of the University Hospital of Larissa. Informed consent was provided by the parents for the underaged healthy individuals and OA patients.

### 2.2. Bone Marrow Mesenchymal Stromal Cells (BM-MSCs) Isolation and Culture

BM-MSCs from young, healthy individuals (*n* = 2) were cultured, reprogrammed to iPSCs (iPSC_N, *n* = 2) and dedifferentiated to iMSCs (iMSC_N, *n* = 2) in a previous study of ours [20]. In the present study, we performed all the above procedures in BM-MSCs derived from OA patients as follows: BM-MSCs from OA patients (MSC_OA, *n* = 5) were cultured in DMEM supplemented with 10% FBS, 1% Pen Strep, 100 mM HEPES, 2 mM L-glutamine, 0.1 mM ascorbic acid, 0.1 mM sodium pyruvate, 2.7 μM L-glucose (DMEM+). All cells were grown in a 37 °C cell culture incubator. Adherent mesenchymal-like cells appeared within the first week of culture. After reaching confluency, they were passaged using 0.05% trypsin (Gibco, BRL) and seeded in new flasks in a split ratio of 1:3. MSCs were fed every 3–4 days and passaged every 4–7 days.

### 2.3. iPSCs Derivation and Culture

MSC_OA were reprogrammed to iPSCs (iPSC_OA, *n* = 5) as previously described [20]. Isolated MSC_OA, 2.5 × 10^4^, were reprogrammed to iPSCs (iPSCs_OA) using a modified synthetic mRNA method [32] (Stemgent, Cambridge, MA, USA). Briefly, 2500 MSCs/cm^2^ were seeded on a feeder layer of irradiated NuFF cells. After two days, repeated transfections of five pluripotency transcription factors (OCT3/4, C-MYC, SOX2, KLF4, LIN28) were initiated and carried out daily using RNAiMAX Transfection Reagent cationic lipid vehicle (Invitrogen, Grand Island, NY, USA). Pluriton Medium (Stemgent) supplemented with B18R interferon inhibitor 200 ng/mL (eBioscience, San Diego, CA, USA) supported reprogramming. After 18 days of transfection, emerged colonies were picked manually for expansion. iPSCs were cultured on Matrigel hESC-qualified Matrix (BD Bioscience, Franklin Lakes, NJ, USA) coated plates in mTeSR1 medium (Stem Cell Technologies, Vancouver, BC, Canada) in standard culture conditions. The medium was changed daily, and cells were passaged every 4–7 days. In vivo, teratoma formation and tumor generation assays to assess the functional pluripotency of iPSCs were done as previously described.

### 2.4. Embryoid Body Differentiation of iPSCs into Induced Mesenchymal Stem Cells (iMSCs) and Expansion

iPSC_OA were induced towards the mesenchymal lineage via Embryoid body (EB) formation [33]. Embryoid bodies were subsequently collected and transferred into gelatin-coated flasks in an MSC differentiation medium consisting of Knockout DMEM supplemented with 10% KSR, 1 mM L-Glutamine, 1× non-essential amino acids and 10 ng/mL bFGF. After the first two passages, iMSC_OA (*n* = 5) were expanded in non-coated flasks in the same conditions as for MSC_OA.

### 2.5. Surface Antigen Expression Analysis

For flow cytometric analysis, iMSC_OA were harvested and incubated with specific MSC-marker antibodies CD90-FITC, CD44-PE, CD105-FITC, CD73-PE, CD29-PE and hematopoietic-marker antibodies CD34 and HLADR. A Cytomics FC500 was used by Beckman Coulter with CXP software for the Cytomics FC500 flow cytometry system version 2,2. We used 400 μL from the total volume of 1 mL iMSC to count the total number of cells and their viability by adding 10 μL of 7-AAD for viable cells, using flow cytometry.

### 2.6. Trilineage Differentiation of iMSC

For induction of trilineage differentiation, iMSC_OA (passage 4) were harvested and reseeded in appropriate concentrations in plates containing MesenCult Osteogenic Stimulatory kit (Stem Cell Technologies, Vancouver, BC, Canada), StemPro Adipogenesis Differentiation kit (Gibco, BRL), StemPro Chondrogenesis Differentiation kit (Gibco, BRL) for osteogenesis, adipogenesis and chondrogenesis respectively and cultured as a monolayer, according to manufacturer’s instructions. Assessment of the differentiated cells was carried out with staining techniques. Alizarid Red and Alkaline Phosphatase stains assessed osteogenesis, Oil Red for adipogenesis and Hematoxylin and Eosin (H&E) for chondrogenesis.

### 2.7. RNA-Sequencing and Differential Gene Expression Analysis

Total RNA was extracted using TRIZOL reagent according to the manufacturer’s protocol and then quantified by the NanoDrop ND-2000 (Waltham, MA, USA). RNA integrity was assessed using Agilent Bioanalyzer 2100 (Santa Clara, CA, USA). Libraries were prepared from 50 ng RNA using the Poly A enrichment method to generate up to 3 million reads per independent library. Sequencing was carried out using the Ion Proton next-generation sequencer (Waltham, MA, USA). Raw data were exported in FASTQ (fq) format, quality control was performed for error rate and GC content distribution, and data filtering was performed to remove low-quality reads or reads with adaptors. The clean reads were mapped to the human reference genome (GRCh38), differential gene expression (DEG) analysis was performed using the DESeq2 method and pairwise gene expression levels were calculated using RPKM (read per kilobase of transcript sequence per millions of base pairs sequenced) value. Fold change (FC) in gene expression was performed on filtered datasets using DESeq2 normalized values. The R_builtin function “cor” was used to compute the Pearson correlation coefficient values between the transcriptomes detected by RNA-seq. DEGs were identified using the R Bioconductor package DESeq2 [34]. The false discovery rate (FDR) was controlled by adjusting the *p*-value using the Benjamini-Hochberg algorithm. Genes with adjusted *p*-value ≤ 0.05 and FC > 1.5 or FC < −1.5 in the final list were set for statistically significant DEGs. The Venn diagrams and heatmaps were generated employing the R/Bioconductor packages “VennDiagram” and “ComplexHeatmap”, respectively [35,36].

### 2.8. miRNA Microarrays and Differential miRNA Expression Analysis

Agilent miRNA Gene Expression 8 × 60 K Microarray (Design ID: SurePrint G3 Human miRNA, miRbase release 21.0, Agilent Technologies, Santa Clara, CA, USA) was used to test the miRNA expression profiling of BM-MSCs, derived iPSCs and iMSCs of both Normal and OA individuals. Sample labeling, microarray hybridization and washing were performed based on the manufacturer’s standard protocols. Total RNA was extracted, measured for quantity and purity, and labeled with Cyanine-3-pCp. The labeled RNAs were hybridized onto the microarray. After washing, the arrays were scanned by the Agilent Scanner G2505C (Agilent Technologies). Microarray data were background-corrected and normalized with quantile normalization using the linear models for microarrays from the R/Bioconductor “limma” package [37]. The corrected and normalized data were initially analyzed with the “limma” package for differential expression. Also, linear models were used to compute *p*-values to determine the significance of the difference between miRNA expression values. The computed differential *p*-values were adjusted in R/Bioconductor with the *p*-adj false discovery rate (FDR) correction algorithm. miRNAs with FDR-corrected *p*-value ≤ 0.05 were considered significantly different. The regulation of these genes was calculated by determining the ratio of fold change. miRNAs with adjusted *p*-value ≤ 0.05 and FC > 1.5 was considered as upregulated, and FC < −1.5 was considered as downregulated.

### 2.9. Functional Enrichment Analysis and Prediction of miRNA Target Genes

Gene Ontology (GO) and Kyoto Encyclopedia of Genes and Genomes (KEGG) pathway enrichment analyses were implemented through the “enrichR” R/Bioconductor package. Functions or pathways that were significantly enriched were identified based on the criterion of *p*-value < 0.05. The enrichment of top GO terms based on FDR-corrected *p*-value was visualized by dot plot analysis. TargetScan [38], miRDB [39] and miRTarBase [40] were used to predict targets of differentially expressed miRNAs. Regulatory networks between differentially expressed miRNAs and their targeted genes were plotted via the “network” package in R and Cytoscape platform (version 3.9.1).

### 2.10. Protein-Protein Interactive (PPI) Network Construction and Module Analyses of DEGs

PPI network for mRNAs was conducted via the Search Tool for the Retrieval of Interacting Genes (STRING) (http://string-db.org (accessed on 23 March 2023); version 11.0). PPI of DEGs was created with a score (median confidence) > 0.4 and was visualized with Cytoscape (version 3.9.1). Molecular Complex Detection (MCODE), a Cytoscape plugin, was implemented to highlight densely connected regions and identify the significant modules through clustering. “MCODE scores ≥ 3,” “k-score = 2,” “Max depth = 100,” “node score cut-off = 0.3,” and “degree cut-off = 2” were the criteria for significant modules. Hub genes were identified from the most significant modules, and their functions were analyzed.

### 2.11. Statistics

All experiments represent biological replicates; technical replicates are repeat tests of the same value. Biological replicates are samples derived from separate sources, such as clones of iPSCs and iMSCs. Statistical comparisons between two groups (N vs. OA) were performed using a two-tailed Student’s t-test for comparing two groups using multiple R/Bioconductor packages. Significant difference was defined with adjusted *p*-values ≤ 0.05. For microarray data analysis, a miRNA with a *p*-value ≤ 0.01 was considered significantly expressed. A gene or miRNA with an adjusted *p*-value ≤ 0.05 was considered considerably different in expression. Functional annotation was considered significant with a *p*-value of ≤ 0.05.

## 3. Results

### 3.1. Characterization of iMSCs Derived from MSCs of OA Patients

**Figure 1:**
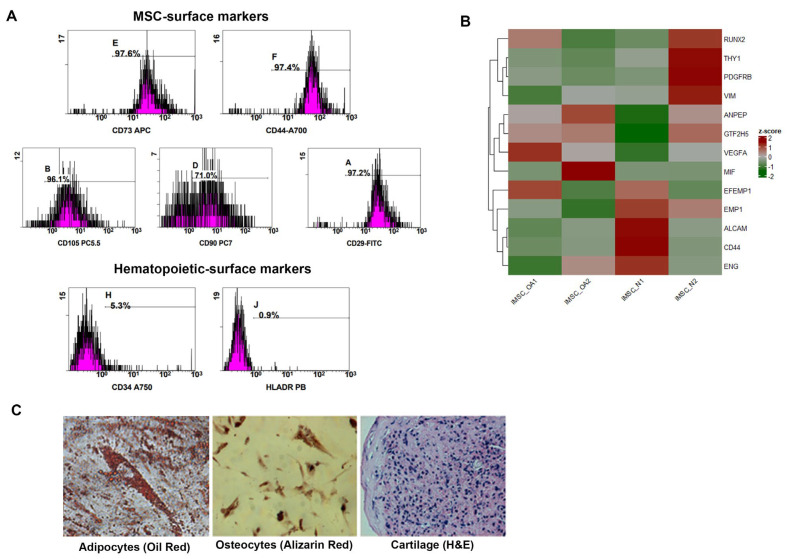
Characterization of iMSCs derived from MSC_OA. (**A**) iMSC_OA expressed all specific surface MSCs markers (CD37, CD105, CD90, CD44, CD29) but not surface hematopoietic markers CD34 and HLADR, as detected by FACS Black: fluorophore-conjugate antibody against surface antigen. Purple: isotype control. (**B**) iMSC_OA expressed MSC-associated markers (*VEGFA*, *Vimentin*, *EMP1*, *PDGFRβ*, *THY1*, *MIF* etc.), as well as differentiation markers (*RUNX2*). (**C**) Confirmation of typical MSC tri-lineage differentiation in iMSC_OA. Oil Red O-positive fat droplets, Alizarin Red-positive calcified matrix and H&E staining were detected in all iMSC_OA under appropriate culture conditions.

iMSC_OA expressed all specific MSCs markers (CD37, CD105, CD90, CD44, CD29) (Figure 1A), MSCs-associated gene markers (*VEGFA*, *Vimentin*, *EMP1*, *PDGFRβ* and *MIF*) and differentiation markers (*RUNX2*) (Figure 1B). Moreover, no expression of hematopoietic markers CD34 and HLADR (Figure 1A) was detected. Oil Red O-positive fat droplets, Alizarin Red-positive calcified matrix and Hematoxylin and Eosin (H&E) staining of cartilage were detected in all iMSC_OA under adipogenic, osteogenic and chondrogenic culture conditions, respectively (Figure 1C). Moreover, to conduct downstream analysis, we also used BM-MSCs isolated from young, healthy individuals (MSC_N), two iPSCs lines (iPSC_N1/2) and iMSCs_N [20].

### 3.2. RNA-Seq Analysis Reveals Differential Gene Expression between MSC_OA and MSC_N

**Figure 2:**
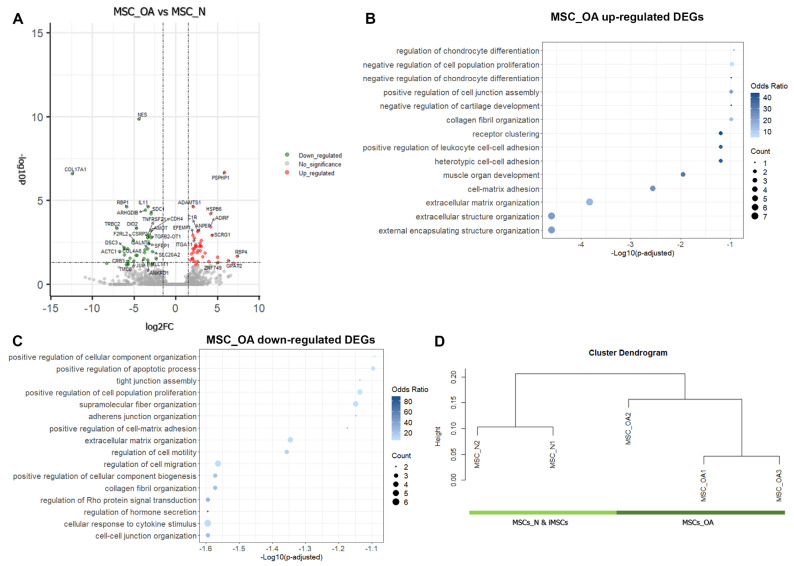
Gene Expression analysis of MSC_OA and MSC_N revealed differences in transcriptome profile. (**A**) Volcano plot of differentially expressed genes (DEGs) between MSC_OA and MSC_N. (**B**,**C**) GO terms of deregulated genes visualized by dot plot with –log10 (*p*-adjusted). (**D**) Clustering analysis of primary MSCs (OA and Normal). As a similarity measure, the Pearson correlation was used.

At first, we performed RNA-seq analysis in BM-MSCs, iPSCs and iMSCs of OA patients and young, healthy individuals. Differential expression analysis of MSCs_N and MSC_OA transcriptome revealed 71 differentially expressed genes (DEGs) between healthy individuals and OA patients (Figure 2A and Table S1). More specifically, in MSCs_OA, 39 genes were up-regulated, and 32 were down-regulated. Gene ontology (GO) and KEGG pathways analysis showed that up-regulated genes in the MSCs_OA group accounted for, amongst others, ECM structure and matrix organization, negative regulation of chondrocyte differentiation and negative regulation of cell proliferation (Figure 2B). In contrast, the 32 down-regulated genes were annotated to GO terms such as tight junction assembly, regulation of cell migration and cell motility regulation and positive regulation of cell-matrix adhesion (Figure 2C), reflecting the heterogeneity that characterizes MSCs. Correlation clustering analysis showed that MSC_OA and MSC_N were divided into two distinct clusters, indicating transcriptomic differences depending on donor-cell condition (Figure 2D).

### 3.3. Transcriptome Landscape Characterization of iPSCs Derived from BM-MSCs of OA Patients and Healthy Individuals Reveals Total Purification of Donor Characteristics

**Figure 3:**
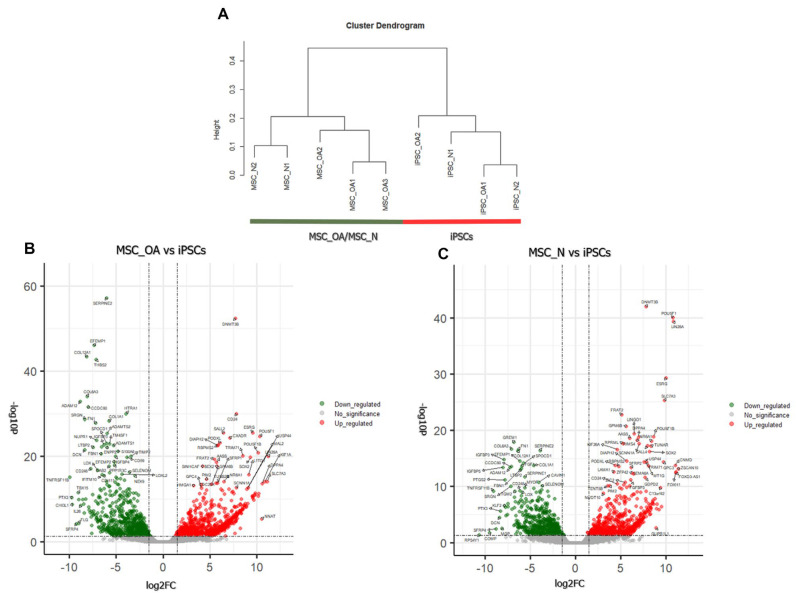
Transcriptome landscape characterization of iPSCs and primary BM-MSCs. (**A**) Clustering analysis of four iPSC cell lines and their primary MSCs. As a similarity measure, the Pearson correlation was used. (**B**) Volcano plot of differentially expressed genes (DEGs) between MSC_OA and iPSCs (**C**). Volcano plot of differentially expressed genes (DEGs) between MSC_N and iPSCs.

**Figure 4:**
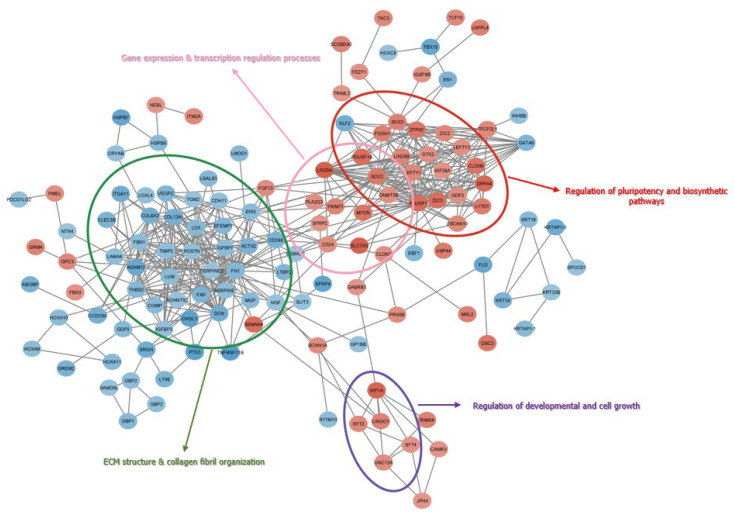
Protein-protein interaction analysis (PPI) for the 200 most deregulated genes in iPSCs compared to MSC_OA Protein-protein interaction analysis (PPI) was performed for the 100 most up-regulated and the 100 most down-related genes in iPSCs compared to MSC_OA, revealed four main interaction clusters. Up-regulated genes (red) in iPSCs were annotated to regulate pluripotency developmental and cell growth processes. Down-regulated genes (blue) in iPSCs were annotated to terms such as ECM organization, skeletal system development and collagen system organization.

Clustering analysis of iPSCs transcriptome (iPSC_OA and iPSC_N) did not show separation by donor-cell derivation (Figure 3A). Subsequent differential gene expression analysis between iPSCs (iPSC_OA and iPSC_N) and MSCs_OA or MSCs_N revealed 1752 and 1090 differentially expressed genes, respectively, among 9856 and 10,838 genes with non-zero counts in the transcriptome data, reflecting about 20% and 10% of the total transcriptomes (Figure 3B,C, Tables S2 and S3). Comparison between all iPSCs with MSC_OA revealed 983 up-regulated genes and 769 down-regulated in iPSCs, while a comparison between iPSCs and MSCs_N revealed 593 up-regulated and 497 down-regulated genes in iPSCs. Next, protein-protein interaction analysis (PPI) was performed for the 100 most up-regulated and 100 most down-related genes in iPSCs compared to MSC_OA (Figure 4). The PPI network revealed four (4) main interaction clusters and GO enrichment and KEGG pathway analysis for these genes determined their molecular and cellular functions. The top 100 up-regulated genes in iPSCs were annotated to regulate pluripotency developmental and cell growth processes. The top 100 down-regulated genes were annotated to terms such as ECM organization, skeletal system development and collagen system organization, all being MSC-associated biological processes (Figure 4).

### 3.4. iMSCs Expression Profiling Reveals Transcriptome Similarities with MSCs Derived from Healthy Young Individuals

**Figure 5:**
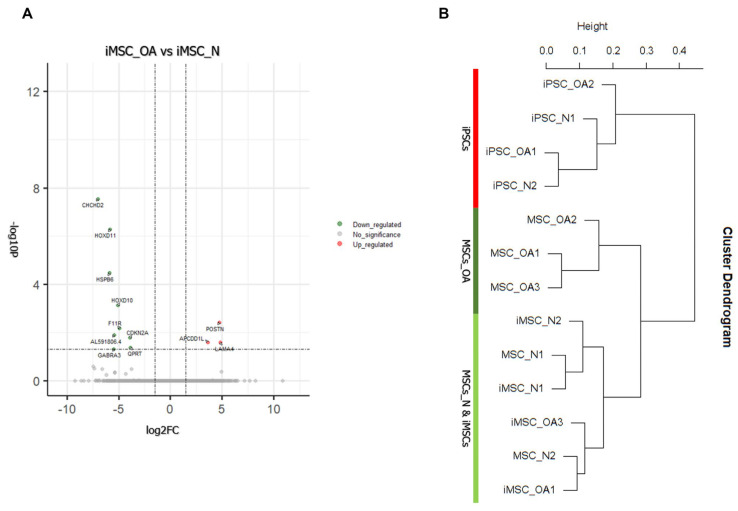
iMSC derived from OA and Normal donors shows similarities with primary BM-MSCs derived from healthy young individuals. (**A**) Volcano plot of differentially expressed genes (DEGs) between iMSC_OA and iMSC_N. (**B**) Clustering analysis of all three cell types: BM-MSCs, iPSCs, and iMSCs. A similarity measure was used, Pearson correlation.

Transcriptome analysis demonstrated that iMSC_OA and iMSC_N exhibited overlapping gene expression patterns, as only 11 genes were differentially expressed between the two groups (Figure 5A). Next, we performed clustering analysis, including MSCs, iPSCs and iMSCs transcriptomes from normal donors and OA patients. We observed two (2) distinct clusters separating MSCs and iMSCs (OA and Normal) from iPSCs. In the main MSCs/iMSCs cluster, two (2) distinct sub-clusters were identified. The first contained primary MSCs derived from OA patients, the second iMSCs (derived from OA and healthy individuals) and primary MSCs derived from young, healthy individuals. The observed distinct clustering indicates transcriptomic similarity between iMSCs and MSCs_N and transcriptomic distance compared to MSCs_OA (Figure 5B).

### 3.5. iMSCs Acquire a Rejuvenated Transcriptomic Profile Indicative of High Proliferation and Differentiation Capability

**Figure 6:**
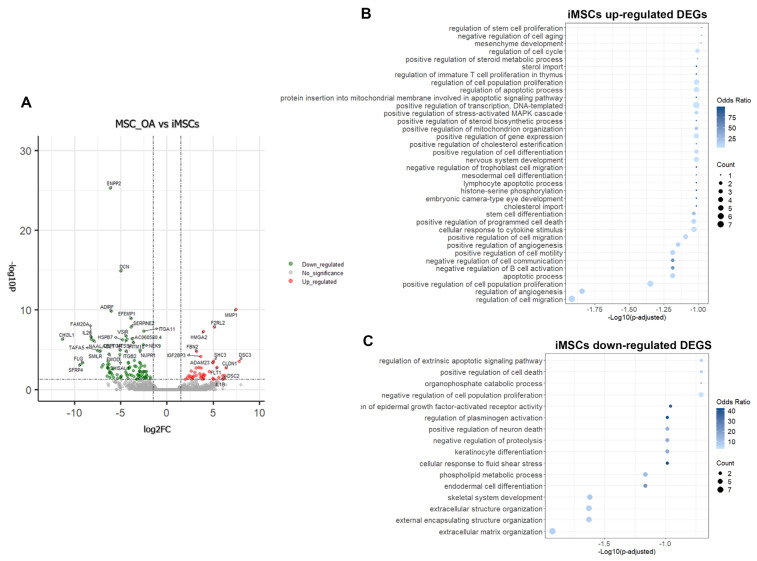
iMSC gene expression analysis reveals a rejuvenated transcriptomic profile. (**A**) Volcano plot of differentially expressed genes (DEGs) between MSC_OA and iMSCs. (**B**,**C**) GO terms of MSC_OA vs. iMSCs deregulated genes visualized by dot plot with −log10 (*p*-adjusted).

**Figure 7:**
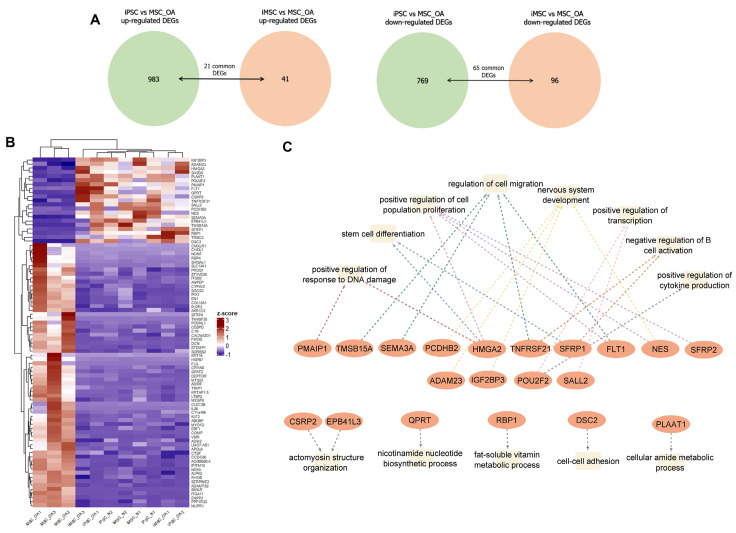
Differentially expressed genes in iMSCs shared the same gene expression profile with iPSCs compared to MSCs_OA. (**A**) Venn diagram of iPSCs vs. MSC_OA DEGs compared to iMSCs vs. MSC_OA DEGs reveals shared genes between iMSCs and iPSCs. (**B**) Heatmap showing clustering of 86 common DEGs between iMSCs and iPSCs compared to primary MSC_OA. (**C**) GO enrichment and KEGG pathway analysis was performed for common up-regulated genes in iPSCs and iMSCs.

Having revealed that iMSCs and MSCs derived from young individuals share common transcriptional patterns, we next prompted to investigate further possible differences at the transcriptome level between iMSCs and primary MSCs_OA. One hundred thirty-seven genes showed differential expression patterns between iMSCs and MSCs_OA (Figure 6A, Table S4). More specifically, 41 genes were up-regulated in iMSCs, annotated to multiple GO biological processes, such as regulation of cell migration and cell population differentiation (Figure 6B), while 96 down-regulated genes annotated to ECM structure organization and skeletal system development pathways (Figure 6C). An interesting finding was that 86 out of the 137 differentially expressed genes in iMSCs shared the same gene expression profile with iPSCs compared to MSCs_OA (Figure 7A,B). We found up-regulated genes, such as *SFRP1/2*, *HMGA2* and *FLT1*, involved in positively regulating cell population proliferation. Moreover, *SEMA3*, *TMSB15* and *TNFRSF21* were engaged in stem cell migration and differentiation pathways and *PMAIP1* in positive regulation of DNA damage response, indicating a “rejuvenation-related” transcriptomic profile (Figure 7C).

### 3.6. iPSCs Express Numerous miRNAs Predicted to Regulate a Pluripotency-Related Target mRNA

**Figure 8:**
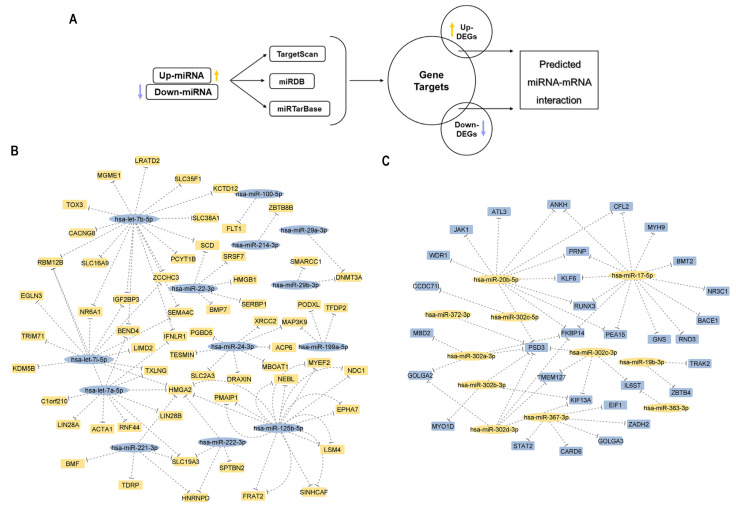
miRNAs predicted to regulate pluripotency-related target genes. (**A**) miRNA target analysis workflow using three different mRNA prediction bioinformatics tools: TargetScan, miRDB and miRTarbase. (**B**) Top 10 up-regulated miRNAs and (**C**) top 10 down-regulated miRNAs in iPSCs potentially interact with deregulated genes in iPSCs compared to MCSs_OA with inversely proportional gene expression pattern (yellow: up-regulated, purple: downregulated).

As previously mentioned iPSCs were characterized by a distinct transcriptome landscape compared to primary MSCs, overexpressing genes important for cell pluripotency. We next investigated whether epigenetic modifiers, such as miRNAs, can influence the iPSCs reprogramming procedure. To evaluate miRNAs that are differentially modulated during MSC reprogramming to iPSC and dedifferentiation to iMSCs, we performed, at first, analysis of miRNA expression profile using miRarrays in MSCs, iPSCs and iMSCs. We next proceeded with a miRNA expression comparison between MSCs_OA and iPSCs. We found 119 mature miRNAs (Table S5) differentially expressed in iPSCs vs. primary MSCs_OA; 68 were up-regulated, and 50 were down-regulated. More specifically, hsa-miR-367-3p, hsa-miR-20b-5p (log2FC = 7.18 and 6.7 respectively)*,* hsa-let-7i-5p and hsa-let-7a-5p (log2FC = −6.68 and −6.25 respectively) were the most deregulated miRNAs in iPSCs. To investigate mRNA gene targets regulated by differentially expressed miRNAs in iPSCs, we conducted miRNA target analysis (Figure 8A) using three different mRNA target prediction bioinformatics tools: TargetScan, miRDB and miRTarbase. Moreover, we further intersected these data with our iPSCs and MSCs_OA transcriptome analysis results and identified 249 DE genes potentially interacting with 97 differentially expressed miRNAs with inversely proportional gene expression patterns (Figure 8B,C, Table S6). GO enrichment analysis was used to group the 97 miRNA-targeted genes and revealed that the predicted targets annotated to multiple GO biological processes, such as regulation of gene expression, cell population proliferation, cell differentiation and biomolecule synthesis (Figure S1).

### 3.7. Predicted mRNA-miRNA Interactions Indicate Potential miRNA Involvement on iMSCs Rejuvenated Transcriptome Profile

**Figure 9:**
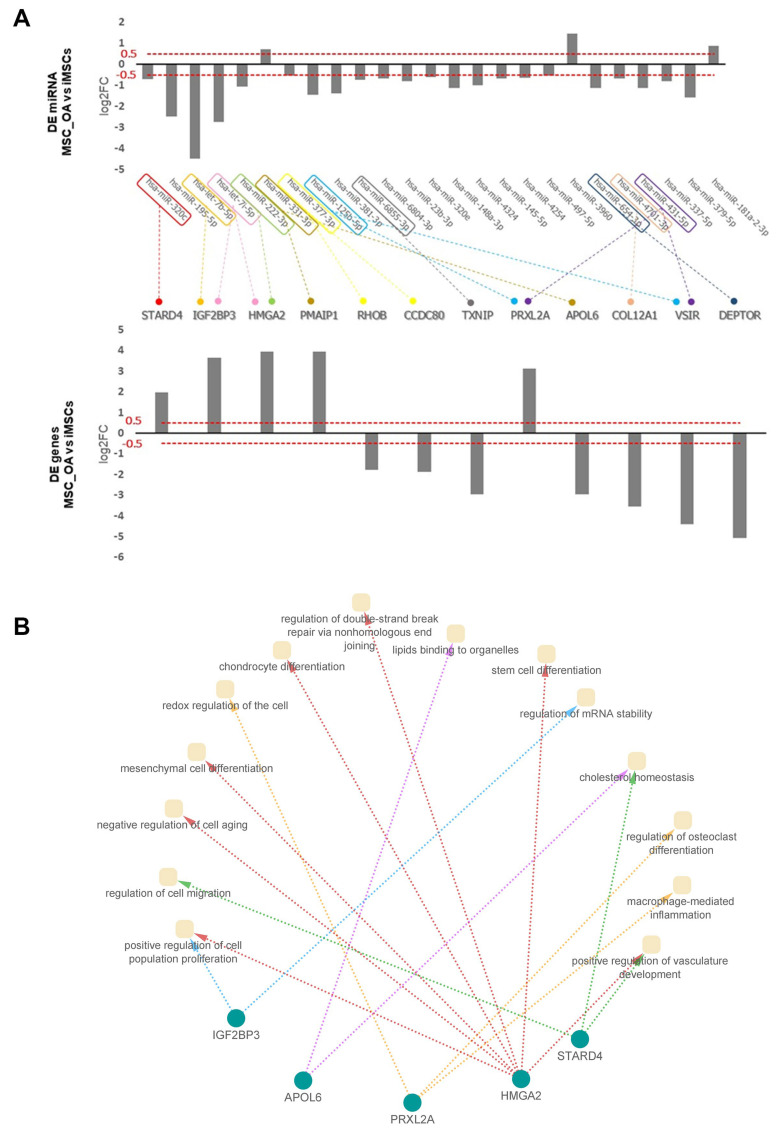
mRNA-miRNA interactions on iMSCs reveal potential regulation of rejuvenated transcriptome landscape. (**A**) Twenty-six mature miRNAs were differentially expressed between iMSCs and primary MSCs_OA, and 12 DE genes potentially interacted with differentially expressed miRNAs. (**B**) GO enrichment and KEGG pathway analysis was performed for 5 of the 12 DE-predicted target genes correlated with DE miRNAs with inversely proportional expression patterns.

Finally, we aimed to explore the involvement of miRNAs in regulating the rejuvenated iMSCs transcriptome profile through possible interactions with their target mRNAs. miRNA microarray data analysis using the *limma* package revealed 26 mature miRNAs differentially expressed between iMSCs and primary MSCs_OA; three (3) out of the 26 were up-regulated, and 23 were down-regulated (Figure 9A). Among them, hsa-let-7b-5p (logFC = −4.46), hsa-miR-195-5p (logFC = −2.46) and hsa-miR-3960 (logFC = 1.47) were the most deregulated miRNAs between these two cell lines. We then identified potential mRNA targets of the DE miRNAs in iMSCs and common mRNAs in the three databases (TargetScan, miRTarbase and miRDB). In accordance with transcriptomic analysis, we also observed that 11 out of the 26 DE miRNAs conserved their expression profile after iPSCs differentiation to iMSCs compared to primary MSCs_OA cells (Table S7). Among them, hsa-let-7b/i expression in primary MSCs_OA reduced after iPSCs reprogramming, and this reduction was also observed during iMSCs differentiation. Moreover, we further intersected microArray data with iMSCs and MSCs_OA transcriptome analysis results and identified 12 DE genes, such as *HMGA2*, *IGF2BP3*, *STARD4*, and *APOL6*, potentially interacting with 15 differentially expressed miRNAs, as hsa-miR-222-3p, hsa-let 7b/I (Figure 9A). An interesting finding was that five of the 12 DE-predicted target genes correlated with DE miRNAs with inversely proportional expression patterns. GO enrichment and KEGG pathway analysis of the five predicted target genes revealed that they annotated to biological processes crucial for mesenchymal stem cell functions and cell therapy applications, such as cell population proliferation, migration, and chondrocyte differentiation (Figure 9B).

## 4. Discussion

Despite MSCs’ large potential for cell therapies in a variety of diseases, their heterogeneity reflecting donor health condition and age, as well as early signs of replicative senescence, limit their expansion in vitro and restrict their use for efficient clinical applications. To overcome the above technical and biological limitations, MSCs derived from pluripotent stem cells (iMSCs) have attracted much attention, as iPSCs represent a reliable and unlimited source to generate homogeneous and well-characterized cell lines [23,41].

In the present study, we analyzed primary BM-MSCs, iPSCs and iMSCs transcriptomes derived from healthy young individuals and OA patients, as well as miRNA-mRNA interactions during these cellular transitions, to understand iMSCs’ gene expression profile regulation, defining thus their properties.

Firstly, we performed RNA-seq analysis of MSCs, iPSCs and iMSCs derived from OA patients and young, healthy individuals. We next proceeded with a gene expression comparison between MSC_OA and MSCs_N. We found that the majority of the differentially expressed genes (DEG) between the two groups were involved in ECM structure organization, cell adhesion and differentiation capabilities, indicating a shift in MSC_OA regeneration properties. This agrees with previous studies that reported changes to MSCs transcriptome patterns between young, healthy, and aged or OA individuals with joint-degenerative disorders, such as osteoarthritis [42], leading to a decline in mesenchymal stem cells’ therapeutic capabilities [43,44,45,46,47,48]. Among the DEG, we found that MSC_OA overexpressed *EFEMP1*, a gene coding an ECM regulation protein that acts as a negative regulator of chondrogenesis by suppressing *SOX9*, type II collagen, and aggrecan expressions [49,50], reducing, therefore, their chondrogenic differentiation potential. Moreover, the observed significant downregulation of collagen XVII (*COL17A1*) and Nestin (*NES*) is indicative of the MSCs_OA senescence state and impaired proliferation and self-renewal ability [51,52,53].

After reprogramming MSC_OA and MSC_N into iPSCs, clustering analysis of iPSCs (iPSC_OA and iPSC_N) did not show separation by donor-cell derivation. Subsequent comparison of gene expression profiles between MSCs (OA and N) and iPSCs revealed distinct transcriptome landscapes for each cell type. PPI analysis performed for the 100 most differentially expressed genes between iPSCs vs. MSC_OA revealed four main interaction clusters. GO enrichment and KEGG pathway analysis for these genes determined that the most up-regulated genes in iPSCs were annotated to the regulation of three germ layers development and cell development, differentiation, and migration, while the most significant KEGG pathways were involved in the regulation of stem cells pluripotency. This confirms the purification effect on donor MSCs transcriptome characteristics and the emergence of distinct gene clusters involved in cell pluripotency, development and growth regulation processes, in agreement with the previously reported biological properties of pluripotent stem cells [54,55]. However, in a very recent study by Khan et al. (2023), it was reported that iPSCs derived from chondrocytes of healthy cartilage exhibited distinct expression signatures that differentiated them from OA-iPSCs, suggesting that iPSCs derived from OA chondrocytes represent features of their physiological origin influencing, therefore, their regenerative capacity [13].

Next, iPSCs dedifferentiation into iMSCs and subsequent comparison of their transcriptome confirmed the high level of similarity between primary MSCs and iMSCs, indicating that although iMSCs originated from iPSCs, they are not pluripotent themselves, being an essential feature for potential use in future clinical applications. Moreover, in accordance with previous reports, we confirmed that all iMSCs shared the same transcriptome “portrait” irrespective of donor condition, overcoming the obstacle of cell source heterogeneity [24,56,57]. Clustering analysis revealed that iMSCs had transcriptomic similarities to MSCs_N rather than MSCs_OA, indicating a “cleansing” of OA-related features. Most of the upregulated genes in iMSCs compared to MSCs_OA were involved in mechanisms of cell migration, proliferation and differentiation, processes that increase the therapeutic potential of iMSCs for OA cell therapy. More specifically, we found that among upregulated genes in iMSCs compared to MSCs_OA were *SFRP1*, *SFRP2* and *SEMA3A*, genes that positively regulate mesenchymal stem cells properties, such as proliferation and chondrogenic differentiation properties through deregulation of *WNT/β-catenin* pathway [58,59,60]. Moreover, *Il-1β* was found upregulated, which is known to increase production of the -also found upregulated -toll-like receptor *CLDN1*, as well as molecules involved in cell migration and adhesion to ECM components, such as collagen and laminin, facilitating, therefore, migration and interaction of cells with extracellular matrix and cartilage [61,62]. In addition, the observed up-regulated *Il-11*, *FLT1*, *HMGA2* and *NES* in iMSCs, compared to MSCs_OA, are all genes involved in the positive regulation of cell proliferation, which play an essential role in stem cell regenerative properties [52,63,64].

However, the ability of MSCs to proliferate and therefore provide high therapeutic potential in stem-cell-based therapies could be associated with the risk of tumorigenesis [65]. In that regard, a wide range of MSCs genes have been shown to play a dual role in regeneration and malignancy formation [66]. In our study, when we compared the transcriptome of MSC_OA vs. iMSCs, we found that *DEPTOR*, *DLX5* and *TIMP1*, all being genes involved in malignancy formation, had decreased mRNA expression profiles in rejuvenated iMSCs.

Another finding in our study was that about 60% of DEG in iMSCs shared the same gene expression profile with iPSCs, indicating the positive effect at the transcriptome level of primary MSCs_OA reprogramming to iPSCs. Among them, we found many genes with increased expression in iMSCs playing essential roles in embryonic tissues and development, indicating that iMSCs possess features associated with early development that could be possibly associated with enhanced regenerative properties. In that regard, a very interesting finding was that among up-regulated genes in iPSCs and iMSCs was *HMGA2*, a gene coding for a chromatin architectural protein expressed at high levels in hESCs and iPSCs and being further transiently up-regulated during the very early stages of hESC/iPSC differentiation [67]. *HMGA2* seems to regulate the balance between self-renewal and differentiation of stem cells [63,68]. In addition, *IGF2BP3*, a downstream target of *HMGA2*, was also found to be up-regulated in iPSCs and iMSCs. *IGF2BP2* is an RNA-binding protein regulating the translation of many mRNAs characterized by high expression during embryogenesis and low expression in adult tissues [69,70], pointing again to features in iMSCs associated with early development. Accordingly, the identified in iMSCs down-regulated genes *EN1*, *CCDC80*, *ENPP2*, *TXNIP* and *EFEMP1*, which are related to loss of aging profiling during iMSCs generation, further point to the rejuvenated features of iMSCs compared to primary MSCs [71,72,73].

So far, we have shown that iMSCs derivation from iPSCs seems to favor the expression of genes conferring high proliferation, differentiation, and migration abilities to iMSCs. The impact of epigenetic regulation on the iMSCs’ “rejuvenated” state has been previously described regarding the DNA methylation profile [25]. However, the role of miRNAs in iMSCs profile is still in its infancy. Therefore, we next aimed to explore, for the first time to our knowledge, the involvement of miRNAs in regulating the “rejuvenated” iMSCs transcriptome profile while revealing their possible interactions with target mRNA molecules.

The involvement of various miRNA families has long been recognized in various developmental processes, including maintaining the cell pluripotency state by targeting several genes implicated in these processes [74]. Comparison of miRarray expression profile between MSCs_OA and iPSCs revealed that several highly expressed DE miRNAs in iPSCs, such as hsa-miR-367, hsa-miR-302b/c/d, hsa-miR-20 and hsa-miR-200 showed similar expression pattern with ES cell lines [75,76], indicating their involvement in pluripotency. Specifically, the observed miR-302-367 cluster is transcriptionally modulated by *OCT4*. It has increased expression in undifferentiated cells like iPSCs, playing a key role in the generation and maintenance of iPS cell-like state [77].

We then explored the involvement of miRNAs in the regulation of iMSCs transcriptome profile. Twenty-six (26) mature miRNAs were significantly modulated in iMSCs compared to primary MSCs_OA, and 11 of them conserved their expression profile after iPSCs differentiation to iMSCs, indicating the reflection of iPSC reprogramming process not only on transcriptome but also on miRNA profile [78]. Specifically, the down-regulated hsa-let-7 in iMSCs, which has been shown to exhibit crucial effects on MSCs regenerative properties, showed reduced expression after iPSCs reprogramming, and this reduction was also observed during iMSCs differentiation [79,80]. In concordance, previous studies have shown that hsa-let7b/i is elevated in aging tissues and participates in multiple pathways regulating the aging process, negatively affecting tissue stem cell function [81].

Moreover, we observed that hsa-let7b/i and target genes *HMGA2* and *IGF2BP3*, both involved in positive regulation of stem cell properties [82], showed an inversely proportional expression pattern, with hsa-let-7b/i decline in iMSCs contributing to the potential enhancement of pluripotency features, such as regulation of specific gene expression, cell population proliferation and biomolecules synthesis. In addition, the higher chondrogenic potential of iMSCs was revealed by the observed down-regulation of hsa-miR-222-3p, which also targets *HMGA2*, pointing towards a chondrogenic potential of iMSCs. This agrees with previous studies, which showed that inhibition of hsa-miR-221-3p, a paralog of miR-222-3p, promotes the formation of cartilaginous repair tissue expressing Collagen type II [83].

Interestingly we also found that hsa-miR-181a-3p, previously shown to be one of the most prominent candidates in the regulation of a balance between chondral and endochondral differentiation of MSCs [84], exhibited significantly increased expression in iMSCs compared to primary MSCs. Hsa-miR-181a has been reported to target and inhibit *RSPO2* resulting in the activation of *BMP* signaling and *SOX9* accumulation, as well as in the inhibition of the canonical *WNT* pathway, affecting the balance between *WNT* and *BMP* signaling to favor chondral outcome [84]. We also observed that the down-regulated hsa-miR-302c and up-regulated hsa-miR-331 potentially target two genes, *STARD4* and *APOL6*, respectively, which have been shown to have a key role in sterol homeostasis, a crucial process for lipid membrane composition as well as vascularization leading to stronger adhesion of cells to ECM components, such as collagen fibrils [85], increasing iMSCs direct interaction with the cartilage.

We believe that the present study points towards the potential of iMSCs for OA cell therapy but also has several limitations. For instance, as there are no previous studies investigating transcriptome-miRnome interactions in iMSCs, future functional studies need to be conducted to investigate in a biological setting the interactions between hsa-let7b/*HMGA2* in chondrogenesis, as well as hsa-miR-302c/*STARD4* and hsa-miR-331/*APOL 6* in lipid metabolism and vascularization. Furthermore, in vivo studies using OA animal models could strengthen and validate our findings regarding the roles of the above-mentioned miRNA-mRNA interactions. This will lead to stronger evidence of the role of iMSCs in OA cell therapy.

## 5. Conclusions

Overall, our data point towards a crucial role of miRNAs in regulating “rejuvenated” iMSCs transcriptome profile through targeting genes implicated in stem cell pluripotent profile. The interactions of miRNAs, as hsa-let7b/i, hsa-miR-221/222-3p, hsa-miR-302c, hsa-miR-181a, hsa-miR-331 with their potential gene-targets *HMGA2*, *IGF2BP3*, *STARD4*, and *APOL6*, if functionally validated, could be proven important players for establishing iMSCs as a source for cell therapy in osteoarthritis.

## Data Availability

Not applicable.

## Supplementary Materials

The following supporting information can be downloaded at: https://www.mdpi.com/article/10.3390/cells12131756/s1, Table S1: Table_S1_DEG_MSC_OA_MSC_N.xlsx; Table S2: Table_S2_DEG_MSC_OA_iPSC.xlsx; Table S3: Table_S3_DEG_MSC_N_iPSC.xlsx; Table S4: Table_S4_DEG_MSC_OA_iMSC.xlsx; Table S5: Table_S5_DE_miRNA_MSC_OA_iPSC.xlsx; Table S6: Table_S6_MSC_iPSC_miRNA_mRNA.xlsx; Table S7: Table_S7_DE_miRNA_MSC_OA_iMSC.xlsx; Figure S1: GO enrichment analysis in iPSCs vs MSC_OA DE-predicted target genes correlated with DE miRNAs.
