# Supplementary figures and images for: Integration of Transcriptome and MicroRNA Profile Analysis of iMSCs Defines Their Rejuvenated State and Conveys Them into a Novel Resource for Cell Therapy in Osteoarthritis
Source: Cells. 2023 Jun 30;12(13):1756. doi: 10.3390/cells12131756 (PMC10340510; doi:10.3390/cells12131756)

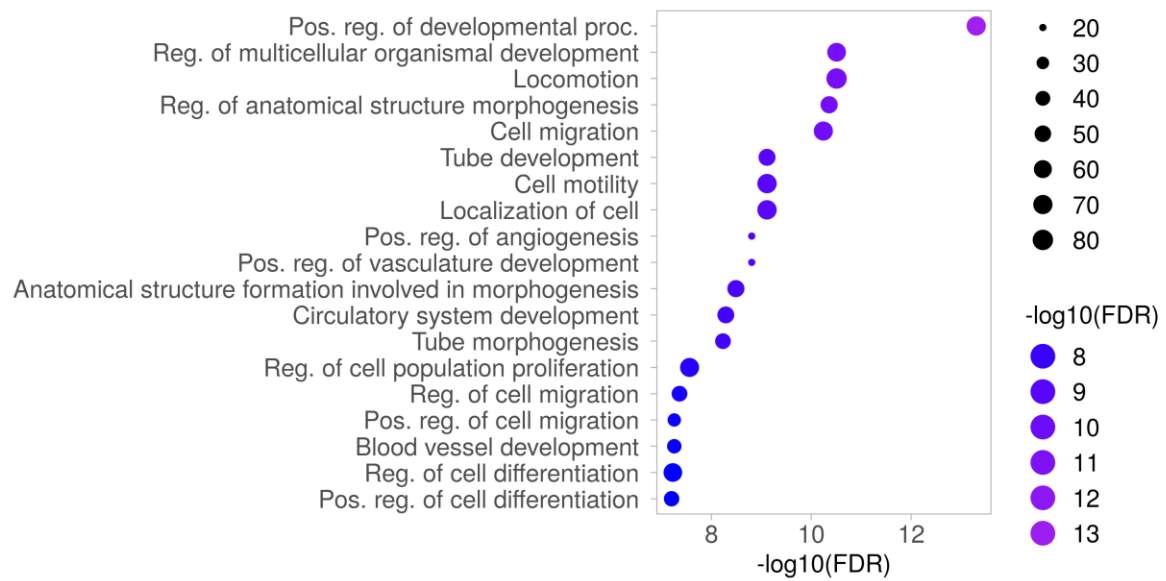

Supplement: Supplementary file 1 [file cells-12-01756-s001.zip › Figure S1.pdf]
